# Interplay between cannabinoids and the neuroimmune system in migraine

**DOI:** 10.1186/s10194-024-01883-3

**Published:** 2024-10-16

**Authors:** Erik Zorrilla, Adriana Della Pietra, Andrew F. Russo

**Affiliations:** 1 Department of Molecular Physiology and Biophysics, University of Iowa, Iowa City, IA 52242 USA; 2 Department of Neurology, University of Iowa, Iowa City, IA 52242 USA; 3 Veterans Affairs Healthcare System, Iowa City, IA 52246 USA

**Keywords:** Neurogenic inflammation, Endocannabinoids, Phytocannabinoids, Synthetic cannabinoids, CGRP, Meninges, Immune cells

## Abstract

Migraine is a common and complex neurological disorder that has a high impact on quality of life. Recent advances with drugs that target the neuropeptide calcitonin gene-related peptide (CGRP) have helped, but treatment options remain insufficient. CGRP is released from trigeminal sensory fibers and contributes to peripheral sensitization, perhaps in part due to actions on immune cells in the trigeminovascular system. In this review, we will discuss the potential of cannabinoid targeting of immune cells as an innovative therapeutic target for migraine treatment. We will cover endogenous endocannabinoids, plant-derived phytocannabinoids and synthetically derived cannabinoids. The focus will be on six types of immune cells known to express multiple cannabinoid receptors: macrophages, monocytes, mast cells, dendritic cells, B cells, and T cells. These cells also contain receptors for CGRP and as such, cannabinoids might potentially modulate the efficacy of current CGRP-targeting drugs. Unfortunately, to date most studies on cannabinoids and immune cells have relied on cell cultures and only a single preclinical study has tested cannabinoid actions on immune cells in a migraine model. Encouragingly, in that study a synthetically created stable chiral analog of an endocannabinoid reduced meningeal mast cell degranulation. Likewise, clinical trials evaluating the safety and efficacy of cannabinoid-based therapies for migraine patients have been limited but are encouraging. Thus, the field is at its infancy and there are significant gaps in our understanding of the impact of cannabinoids on immune cells in migraine. Future research exploring the interactions between cannabinoids and immune cells could lead to more targeted and effective migraine treatments.

## Introduction

Migraine is a multifaceted neurovascular disorder burdening up to 15% of the world population with a great effect on life quality and global economy [1]. Despite being ranked as the second most disabling disease in the world [2], migraine treatment availability remains insufficient. Indeed, even the recent CGRP monoclonal antibodies still leave roughly 50% of migraine patients untreated [3–5]. Therefore, innovative therapeutics and targets are needed.

**Fig. 1:**
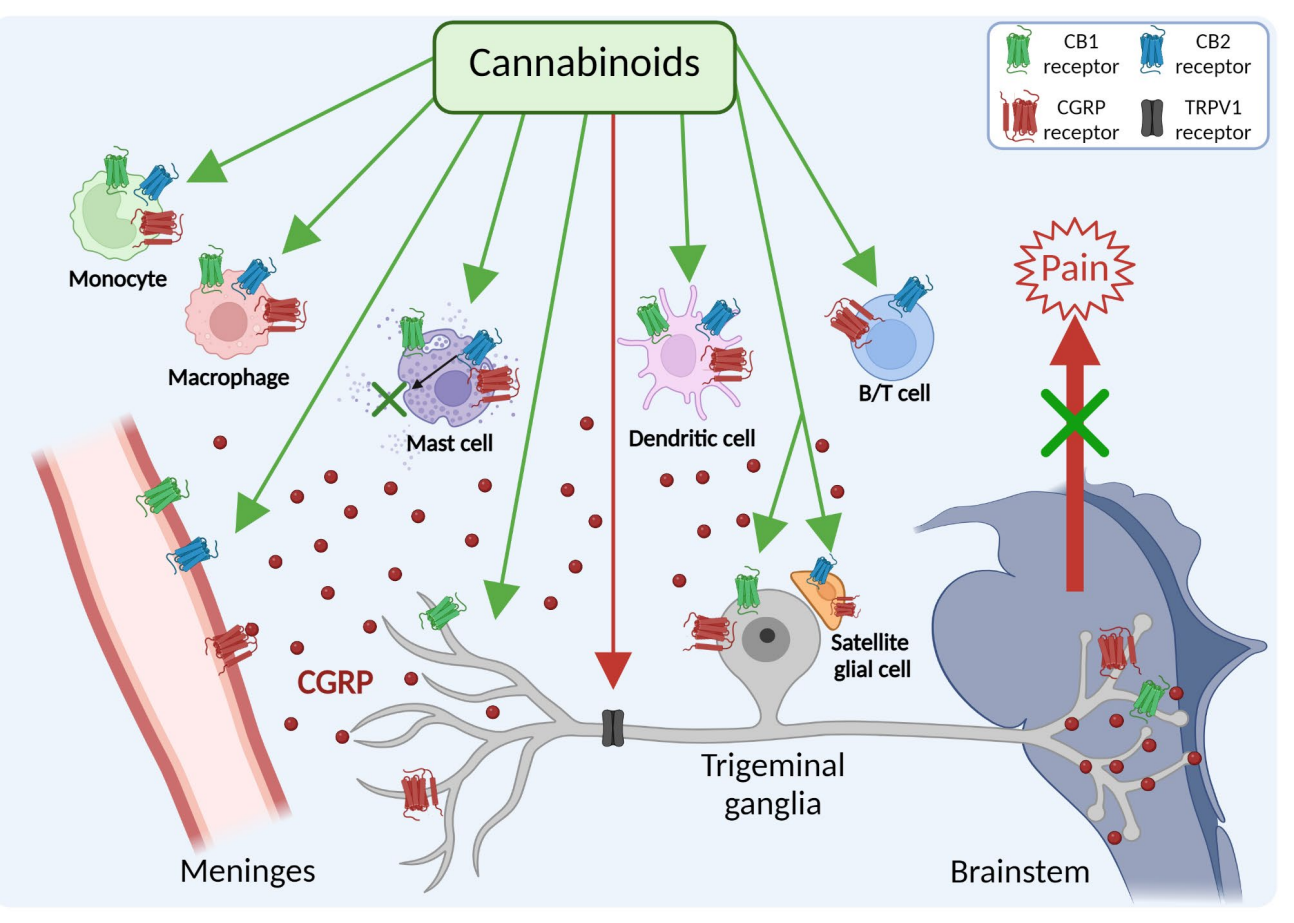
**Potential targets of cannabinoids in the trigeminovascular system** Cannabinoids can interact with CB1 and CB2 receptors in trigeminal ganglia neurons, satellite glia, blood vessels, and meningeal immune cells, as well as trigeminal TRPV1 receptors. For clarity, a generic trigeminal neuron is represented and not all cell types in the trigeminovascular system are shown. Potential analgesic targets for cannabinoids are shown in green arrows. A pathway that could increase nociception via the TRPV1 receptor is shown in red. Release of CGRP from trigeminal nerve fibers and within the ganglia is shown. CGRP can contribute to neurogenic inflammation by degranulation of mast cells, dilatation of blood vessels, and possibly acting on immune cells. The CGRP receptor icon is meant to represent both canonical and AMY_1_ receptors

As a starting point for identifying new targets, this review will focus on immune cells in the trigeminovascular system, which is generally recognized to play a key role in migraine pain [6]. The trigeminovascular system is composed of afferent trigeminal nerve fibers in the meninges that innervate blood vessels and immune cells, cell bodies in the trigeminal ganglia (TG), and efferent central connections in the trigeminal cervical complex in the medulla [6–8]. During a migraine attack, calcitonin gene-related peptide (CGRP) is released from trigeminal sensory fibers and ganglia, which can promote neurogenic inflammation and nociceptive signals that are relayed to the brainstem and higher brain regions to generate the perception of pain (Fig. 1) [7, 9, 10]. Within the trigeminovascular system, as shown in Fig. 1, CGRP receptors are present on meningeal blood vessels, glia, and trigeminal ganglia neurons [11]. In addition to the canonical CGRP receptor, a second CGRP receptor, AMY_1_, is also present on trigeminal ganglia neurons [12–14]. Immunohistochemical and RNA evidence for canonical receptor subunits (CLR and RAMP1) and AMY_1_ receptor subunits (CTR and RAMP1) has been reported by several groups [12–18]. Importantly, a host of immune cells, including macrophages, monocytes, mast cells, dendritic cells, B cells, and T cells are known to express CGRP receptors and these cells are also present within the meninges and macrophage-like cells are also present in the ganglia (Fig. 1) [8, 11, 19].

While not the main focus of this review, glial cells can also play roles in the immune system [20], including release of inflammatory cytokines and central sensitization [21], which are both relevant to migraine. Of note, glia can express class II MHC molecules, as more commonly associated with B cells, macrophages, and dendritic cells. MHC expression in astrocytes and microglia can be induced or regulated by cytokines, neurotransmitters, and neuropeptides [22], such as might occur during a migraine attack. Beyond inflammatory events, there is disrupted astrocytic clearance of glutamate in familial hemiplegic migraine type 2, which leads to increased cortical neurotransmission [23]. In addition, the glymphatic system is an astrocyte-associated lymphatic system in the brain that is another area of possible glial involvement in migraine [24].

In migraine, CGRP is generally considered to be pro-inflammatory, but it can also have anti-inflammatory actions depending on the tissue microenvironment and specific target cells [11, 25]. In the skin, CGRP release following nociceptor stimulation promotes the recruitment and activation of immune cells and the production of proinflammatory cytokines in cutaneous inflammation [26]. Injection of CGRP into the rat TG has been shown to increase IL-1β mRNA and CGRP increased the release of IL-1β and other cytokines from cultured satellite glial cells (SGC) [27]. Similarly, injection of CGRP into the temporomandibular joint stimulated expression of proteins in the TG associated with peripheral and central sensitization and activated astrocytes and microglia in the trigeminal nucleus caudalis [28]. Another study found that intracisternal injection of CGRP increased pain responses and astrocyte activation, but not microglial activation [29]. In contrast, it has recently been shown that injection of CGRP into the rat TG shifts the polarity of macrophages to an anti-inflammatory M2 phenotype [30]. Thus, CGRP is likely to play a complex role in the neurogenic inflammatory process in migraine.

Nociceptors, glia, and immune cells are responsible for the neuroimmune communication, involving a repertoire of inflammatory mediators, such as cytokines, chemokines, and Toll-like receptors. In particular, Toll-like receptors, which have been traditionally found on innate immune cells, are also expressed in nociceptive neurons in both dorsal root ganglia and TG where they help regulate sensory functions, such as pain and itch [27, 31]. Additionally, the degranulation of mast cells requires interaction between mast cells and peripheral nerves, which is mediated by the calcium-dependent cell adhesion molecule N-cadherin [32]. Notably, a weak mast cell degranulating effect of CGRP indicated by histamine release was found in rat dura mater but not in the human dura [33, 34]. As mentioned above, in addition to mast cells, macrophages, dendritic cells, B cells, and T cells, have been reported to express the CGRP receptor [11, 19, 35], allowing CGRP to act as immunomodulator.

Dural stimulation with inflammatory mediators, such as inflammatory soup and complete Freund’s adjuvant (CFA), has been extensively studied in animal models to understand its effects on CGRP and related pain mechanisms [36, 37]. Application of CFA to the dura mater in rats resulted in increased CGRP-positive fibers in the trigeminal ganglion, correlating with periorbital allodynia [38]. Another study done on rats showed that inflammatory soup application led to heightened levels of CGRP and other sensitization markers in the trigeminal nucleus caudalis [39], and changes in locomotor behavior in another study done in mice [40], suggesting a possible link between inflammation and migraine-like symptoms.

Consistent with a possible meningeal neuroimmune interaction in migraine, cortical spreading depression is also able to activate meningeal macrophages and dendritic cells [41]. Another classical migraine trigger nitroglycerin (NTG) is also able to activate dural immune cells. Indeed, NTG caused delayed meningeal inflammation such as mast cell degranulation, macrophage activation, NF-κB-dependent upregulation of inducible nitric oxide synthase (iNOS), and the appearance of interleukin (IL)-1 beta (IL-1β) and IL-6 [42, 43]. Once activated, there is a feedback activation of neurons as the immune cells release lipids, cytokines, and growth factors that can play a key role in sensitizing nociceptor sensory neurons [44–47]. In this review we will address how cannabinoids might modulate this neuroimmune cycle in migraine.

Cannabinoids have attracted interest as potential anti-migraine compounds [48, 49]. Despite its side effects, medical marijuana has been reported to reduce migraine frequency attacks [50]. Traditionally, within the primary trigeminal nociceptive afferents, the activation of CB1 receptors by endogenous cannabinoids typically leads to the suppression of CGRP release from peripheral terminals [51–53]. Conversely, within central processes, endocannabinoids act to inhibit glutamate release, which in turn modulates the transmission of nociceptive signals to second-order neurons in the brainstem [51]. Apart from interacting with the conventional inhibitory CB1 and CB2 receptors, endogenous cannabinoids can also participate in neuromodulation mediated by non-cannabinoid receptors. For instance, it has been shown that anandamide (AEA) can activate the transient receptor potential vanilloid receptor (TRPV1) at elevated concentrations (Fig. 1). This activation might lead to the release of CGRP and facilitate nociceptive signaling [52, 54]. In this regard, the potential role of TRPV1 in migraine has been recently reviewed [55, 56]. However, the TRPV1 antagonist SB-705,498 showed no benefit in a clinical migraine study, highlighting that therapies targeting this receptor alone are insufficient for effectively treating migraine symptoms [57].

Meningeal immune cells express both cannabinoid receptors CB1 and CB2 with higher prevalence of CB2 (Fig. 1) [58]. Therefore, immune cells represent potential targets for an innovative migraine cannabinoid treatment through neuroimmune modulation. This review aims to evaluate recent studies on the efficacy and underlying mechanisms of cannabinoid-based treatments for migraine, with a particular emphasis on their interactions with immune cells.

## Cannabinoids and migraine

Cannabis has been historically used for managing migraine symptoms [59, 60]. Despite potential negative psychoactive side-effects of cannabis, its derivative compounds (cannabinoids) have emerged as a new class of analgesic and anti-migraine agents. The endocannabinoid system (ECS) is a complex molecular system maintaining homeostasis and it is involved in controlling many pathological processes, including nociception [61]. The ECS is composed of the main endocannabinoids, AEA and 2-arachidonoylglycerol (2-AG), their synthesizing and degrading enzymes and their class A G-protein coupled receptors CB1 and CB2, which mostly activate the inhibitory Gα_i/o_ G proteins [62–65]. CB1 receptors are expressed at higher levels in both the central nervous system (CNS) and peripheral nervous system (PNS) where their activity regulates excitability and neurotransmission. In contrast, CB2 receptors mostly modulate immune responses, in meningeal immune cells and microglia [62, 66, 67].

**Fig. 2:**
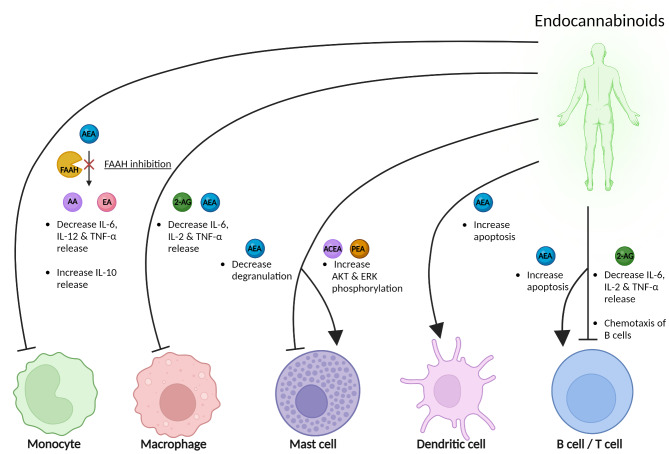
**Endocannabinoids and immune cells** The effects of endocannabinoids on immune cells are indicated. Inhibition of FAAH leads to an increase of anti-inflammatory cytokine IL-10 and a reduction of proinflammatory cytokines IL-6, IL-12 and TNF-α in monocytes. Similarly, 2-AG and AEA reduce the release of IL-6, IL-2, and TNF-α in macrophages. AEA inhibits degranulation in mast cells, while ACEA and PEA enhance the phosphorylation of AKT and ERK, indicating activation of these signaling pathways in mast cells. AEA also promotes apoptosis in dendritic cells, contributing to immune regulation. Notably, 2-AG induces chemotaxis in B cells and similarly decreases the release of IL-6, IL-2, and TNF-α in both B and T cells, highlighting its role in immune cell migration and cytokine regulation

Endocannabinoids degrading enzymes monoacylglycerol lipase (MAGL) and fatty acid amide hydrolase (FAAH) have been found distinctly active in the main areas involved in migraine pain signaling [68]. Apart from their degradation, endocannabinoids levels are maintained physiologically low via alternative sequestration mechanisms involving fatty acid binding proteins [69], heat shock proteins [70], sterol carrier protein 2 [71] located in lipid rafts [72], or bidirectional membrane transporters [73]. Therefore, it is hypothesized that endocannabinoids are released on demand based on neuronal activity to counteract a pathological condition [74]. Increased AEA levels have been found in rat meninges under nociceptive stimuli [48]. FAAH, 2-AG and AEA regulate immune responses by reducing the release of IL-6, IL-2, and TNF-α in monocytes, macrophages, B, and T cells, while AEA inhibits mast cell degranulation, promotes apoptosis in dendritic cells, and compounds like arachidonyl-2’-chloroethylamide (ACEA) and palmitoylethanolamide (PEA) activate signaling via AKT and ERK phosphorylation in mast cells, with 2-AG also inducing chemotaxis in B cells (Fig. 2). Overall, the enhancement of the endocannabinoids and/or their signaling via endocannabinoid receptors in the areas surrounding meningeal afferents, can potentially reduce the generation and transmission of pain to the second order brainstem neurons [75].

**Fig. 3:**
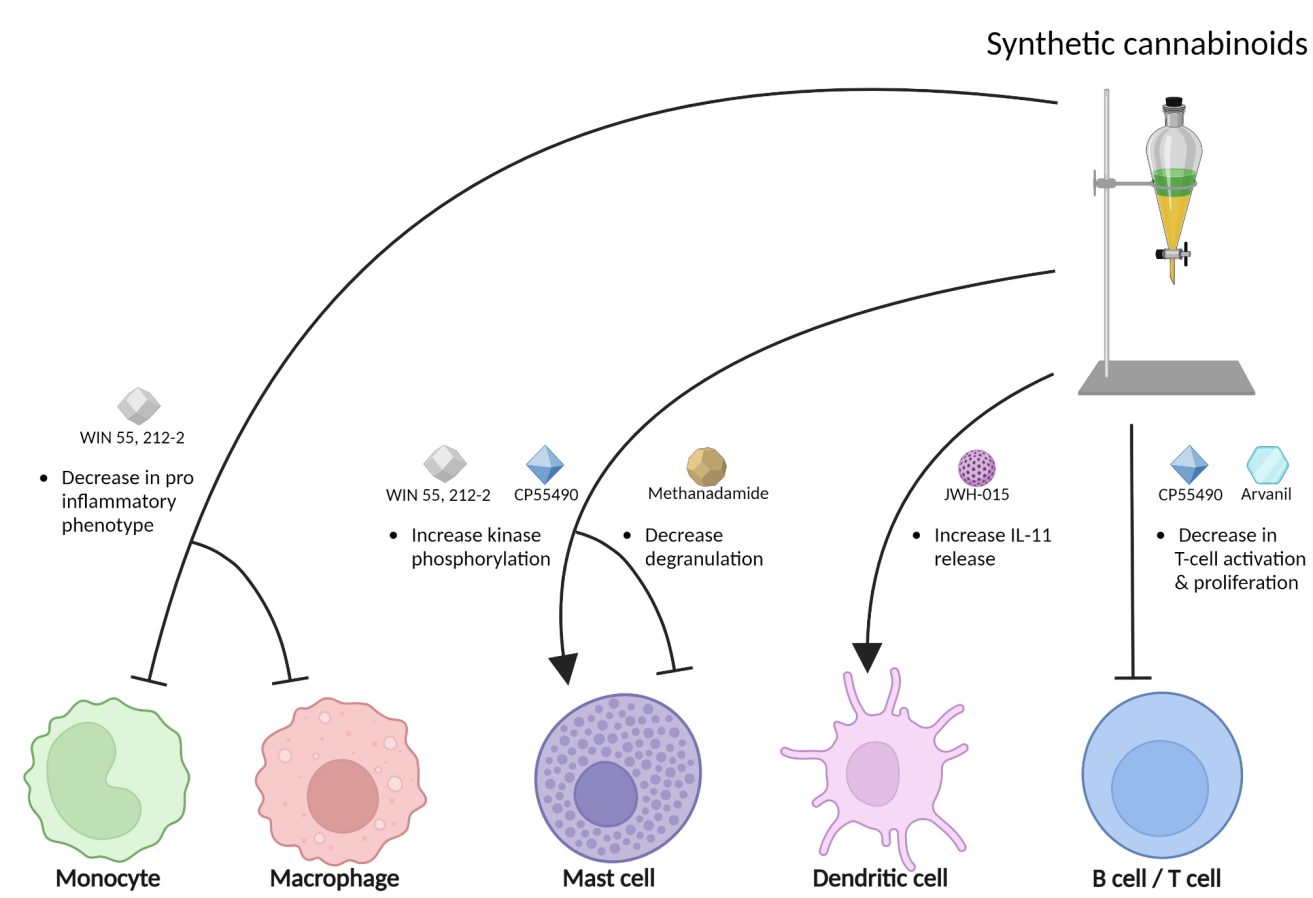
**Synthetic cannabinoids and immune cells** The effects of synthetic cannabinoids on immune cells are indicated. WIN 55, 212-2 mitigates the pro-inflammatory phenotype in monocytes and macrophages, contributing to a reduced inflammatory response. Both WIN 55, 212-2 and CP55490 enhance kinase phosphorylation in mast cells, which may signal cellular activation or functional modulation. Methanandamide decreases degranulation in mast cells, potentially reducing inflammation. JWH-015, a CB2 receptor agonist, is shown to stimulate the release of the anti-inflammatory cytokine IL-11 in dendritic cells, further supporting its role in immune suppression. Lastly, CP55490 and arvanil effectively decrease the activation and proliferation of T cells, indicating potential therapeutic applications in managing T cell-mediated disorders

Synthetic cannabinoids are laboratory-made compounds chemically resembling phytocannabinoids, e.g. nabilone, WIN 55,212-2 and HU-210, binding as full agonists to CB1 and CB2 receptors [76, 77]. A study done on rats showed that administration of WIN 55 212-2 was successful in alleviating NTG induced hyperalgesia through its actions on both CB1 and CB2 receptors [78]. A clinical trial where nabilone was given to treat medication overuse headache showed a reduction in pain intensity, analgesic intake, and improving quality of life in migraine patients, with mild and sporadic side effects [79]. Additionally, it has been demonstrated that peripherally restricted synthetic cannabinoids prevent sensitization of trigeminal neurons in mouse models of migraine, which potentially offers a therapeutic approach with minimal central nervous system side effects [80]. Synthetic cannabinoids like WIN 55, 212-2, CP55490, methanandamide, JWH-015, and arvanil modulate immune cell functions by reducing the pro-inflammatory phenotype in monocytes and macrophages, enhancing kinase phosphorylation in mast cells, decreasing mast cell degranulation and T cell activation, and stimulating the release of anti-inflammatory cytokine in IL-11 in dendritic cells, highlighting their potential in managing inflammatory and T cell-mediated disorders (Fig. 3). However, synthetic cannabinoids are still widely regarded as hazardous and toxic and warrant further research to determine their applicability in treating migraine and other neurological disorders [77].

**Fig. 4:**
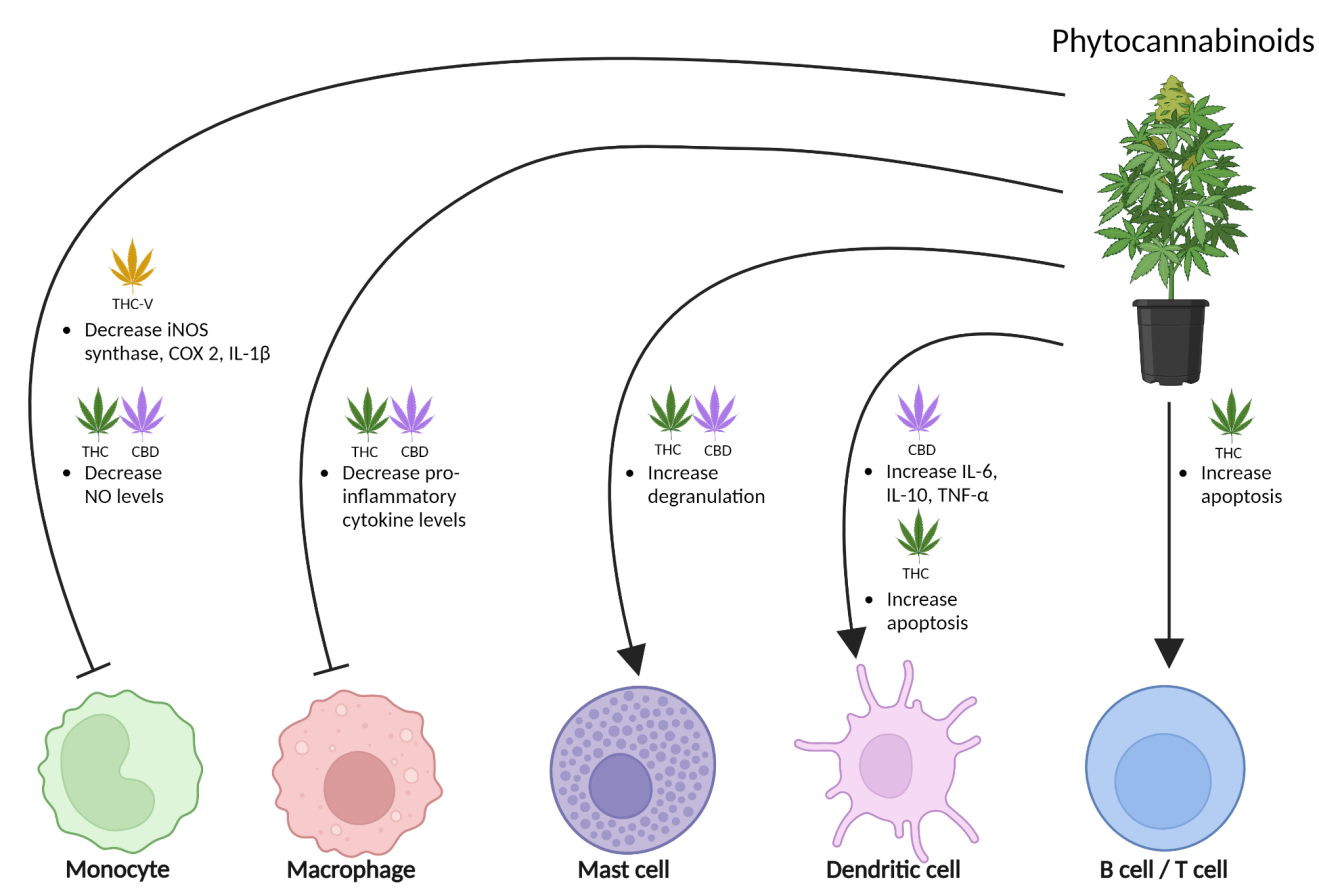
**Phytocannabinoids and immune cells** The effects of phytocannabinoids on immune cells are indicated. THC and CBD both reduce NO levels in monocytes, which may diminish their inflammatory capabilities. THC-V, is shown to decrease iNOS, COX-2, and IL-1β in monocytes, suggesting a broad anti-inflammatory action. In macrophages, THC and CBD lower pro-inflammatory cytokine production, further emphasizing their potential to mitigate inflammatory responses. Contrarily, THC and CBD enhance degranulation in mast cells, which could potentially increase inflammatory responses, depending on the context. THC induces apoptosis in dendritic cells, potentially affecting antigen presentation and immune tolerance. CBD, interestingly, increases the levels of both pro-inflammatory (IL-6, TNF-α) and anti-inflammatory (IL-10) cytokines in dendritic cells, indicating a complex regulatory role. Additionally, THC promotes apoptosis in both B and T cells, which may contribute to its immunosuppressive effects

Phytocannabinoids, including Δ9-tetrahydrocannabinol (THC) and cannabidiol (CBD), are found in the cannabis plant [81]. THC is a partial agonist of the cannabinoid receptors with high affinity at CB1 and CB2 receptors [62], binding the same site as AEA and 2-AG. Instead, CBD mostly targets CB1 by acting as a negative allosteric modulator and activates CB2 as a partial agonist [82, 83]. At very high concentrations, CBD can activate CB1 at orthosteric binding sites, while standard CBD concentrations cannot activate CB1 by themselves, acting allosterically [83]. Therefore, while the combination of CBD and THC activates cannabinoid receptors, at the same time, CBD can strongly attenuate THC effects, protecting against psychotropic side effects [84]. CBD can also inhibit AEA reuptake and its degradation by FAAH, promoting the endocannabinoid system over THC effects [85]. Ideally, a similar beneficial effect could be obtained by replacing the psychoactive THC with endocannabinoid enhancement, representing a chance to manage migraine pain. Overall, phytocannabinoids THC and CBD modulate immune functions by reducing inflammatory markers in monocytes and macrophages and altering cytokine levels in dendritic cells, with tetrahydrocannabivarin (THC-V) enhancing these anti-inflammatory effects; however, both also increase mast cell degranulation and promote apoptosis in dendritic, B, and T cells, showcasing a complex regulatory role on immune responses (Fig. 4).

There is a growing body of preclinical and clinical research that suggests cannabinoids can decrease the frequency and severity of migraine symptoms [86–88]. Additionally, “clinical endocannabinoid deficiency” has been implicated in several neurological disorders, including migraine [89, 90]. Compared to non-migraine control subjects, lower levels of AEA are seen in the cerebrospinal fluid [91] and platelets [92] of patients suffering from chronic migraine. This decrease of AEA is thought to play a role in increased production of CGRP and nitric oxide (NO) [91], both of which are implicated in migraine pathogenesis [93, 94]. Moreover, a decreased expression of CB1 receptors has been associated with predisposition to migraine [90]. Along those lines, cannabinoid receptors are found on cells in both the CNS and PNS including immune cells [62]. The role these receptors play in modulating immune cell function merits further investigation, particularly in the context of migraine.

## The immune system and migraine

The immune system plays a critical role by defending the body but is also involved in various neurological conditions, including migraine [95]. The immune system is a complex network of cells, tissues, and organs that normally defends the body against harmful invaders such as pathogens, toxins, and abnormal cells. It consists of two main components: the innate immune system and the adaptive immune system. The innate immune system provides immediate defense using physical barriers and cells like monocytes, macrophages, dendritic cells, and mast cells that engage in phagocytosis, produce cytokines, and trigger inflammatory responses [96]. On the other hand, the adaptive immune system, featuring B cells and T cells, offers a specialized response through memory cells that effectively recognize and combat specific pathogens [96, 97].

An important role of immune cells is an intrinsic response called neurogenic inflammation [98]. Neurogenic inflammation involves neuronal activation of immune cells and glia to release inflammatory mediators that impact many functions [99]. One hypothesis is that during a migraine attack, immune cells may become activated and release inflammatory mediators that exacerbate symptoms [19, 100]. In support of this prediction, a study reported reduced total T-cell counts and alterations in regulatory T-cell profiles in the blood of migraine patients, indicating potential immune dysregulation [101]. However, a large population-based study found no statistically significant associations between blood-based immune markers and migraine, suggesting that immune cell counts may not influence migraine susceptibility [102]. Indeed, measurements of pro-inflammatory cytokines in migraine patients have yielded conflicting results with respect to changes during the attack and comparison to control subjects, as recently reviewed by several groups [103–105]. This variability highlights the need for further research to better understand the role of inflammation across different migraine subtypes and phases but leaves open the possibility that targeting immune cells might offer new strategies for managing migraine symptoms.

## Cannabinoids targeting immune cells for migraine treatment

Cannabinoids have shown efficacy in reducing neurogenic inflammation in preclinical models of migraine [52, 54, 106–108]. Specifically, both the endocannabinoid AEA and the synthetic cannabinoid WIN55, 212-2 reduced dural inflammation through CB1 activation [54, 106]. However, it is important to consider the role of AEA in nociception as a TRPV1 agonist. Indeed, it has been shown that endo-vanilloids can activate trigeminal afferents expressing functional TRPV1 receptors, triggering CGRP release [52, 109]. Moreover, it is known the immunosuppressive role of endocannabinoids on immune cells, which is primarily attributed to their interaction with CB2 receptors, leads to the inhibition of the cAMP/protein kinase A pathway [110]. This results in decreased expression of cAMP-responsive genes. Additionally, endocannabinoids exert effects at the nuclear level, such as phosphorylation of IκB-α, which enhances the transcription of several apoptotic genes regulated by NF-κB [111]. They also activate peroxisome proliferator-activated receptor gamma, which in turn inhibits nuclear factor of activated T cells, and disrupt the cell cycle by activating p21waf-1/cip-1 and inducing G1/S phase arrest [58]. However, while the effects of endocannabinoids on meningeal nerve terminals in migraine nociception have been studied [112], the extent of various cannabinoids’ impact on these terminals and on their crosstalk with the surrounding immune cells remains poorly investigated. The actions of cannabinoids in specific immune cells that may be relevant to migraine are described below and summarized in Table 1.

**Table 1:** Effects of cannabinoids on immune cells and their evaluation in migraine-related studies

Immune cell type	Cannabinoid tested	Effect	Experimental system	Reference
Monocytes	WIN55,212-2	⇓ Differentiation into pro-inflammatory phenotype (LPS treated)	Cell culture	[118]
	THC	⇓ NO pro-inflammatory cytokine levels (LPS treated)	Colon tissue	[119]
	CBD			
	THC-V	⇓ iNOS synthase, COX-2, IL-1β levels (caused by LPS)	Cell culture	[120]
	FAAH inhibition	⇓ IL-6, IL-12, TNF-α ⇑ IL-10 (caused by LPS & IFN-γ)	Cell culture	[121]
Macrophages	WIN55,212-2	⇓ Differentiation into pro-inflammatory phenotype (LPS treated)	Cell culture	[118]
	AEA	⇓ IL-6 and NO in dose dependent manner in response to LPS	Cell culture	[122]
	THC			
	2-AG	⇓ IL-6 and ⇑ NO		
	THC	⇓ IL-6, IL-1β, TNF-α in response to LPS (CB2 mediated)	Cell culture	[123]
	CBD			
Mast cells	PEA	⇑ AKT & ERK phosphorylation via CB2. ⇓ Secretion via CB1. ⇓ NGF release via GPR55.	Cell culture	[130, 132]
	ACEA			
	WIN55,212-2			
	CP55940			
	AEA	⇓ SP-induced human mast cells degranulation	Cell culture	[131]
	CBD	⇑ Degranulation	Cell culture	[118]
	THC			
	Methanandamide	⇓ Degranulation	NTG rat migraine model	[134]
Dendritic cells	AEA	⇑ Apoptosis via NF-κB pathway	Cell culture	[111]
	THC			
	CBD	⇑ release of IL-6, TNF-α, IL-10 in response to LPS	Cell culture	[140]
	JWH-015	⇓ replication of infected cells ⇑ IL-11 release	Cell culture	[142]
B cell/ T cell	2-AG	Chemotaxis of B cells	Cell culture	[143]
		⇓ IL-6, IL-2, TNF-α release	Mice with methylated BSA antigen	[152]
	Arvanil	⇓ T cell activation	Cell culture	[153]
	AEA	⇑ Apoptosis ⇓ T cell proliferation	Cell culture	[177]
	THC	⇑ Apoptosis		
	CP55490	⇓ T cell proliferation		

## Macrophages, monocytes, and cannabinoids

Macrophages and their precursor monocytes are phagocytic immune cells that serve protective roles against pathogens and cancer cells in the body [113]. Mouse studies have shown that macrophages may contribute to neuronal sensitization in migraine by potentially creating a local environment that predisposes to pain onset through interactions with sensory neurons [114]. Moreover, migraine patients exhibit altered levels of circulating CD14, TNF-α, and MIP-1, with increased serum CD14 concentrations and decreased TNF-α expression in monocytes during the interictal period [115]. Furthermore, monocytes from migraine patients exhibit higher levels of NO production and prostaglandin E2 release, potentially contributing to neurovascular changes leading to migraine attacks [116]. These findings highlight the involvement of monocytes and macrophages in migraine.

Cannabinoids have been shown to modulate the function and differentiation of monocytes. Both cannabinoid receptors have been found on macrophages and monocytes [117]. Specifically, cell culture studies suggest that WIN55,212-2, THC and CBD can inhibit monocyte activation, reduce cytokine secretion, and alter the differentiation of monocytes into dendritic cells [118, 119]. THC-V, a derivative of THC, effectively reduces the levels of iNOS, cyclooxygenase-2 (COX-2), and IL-1β in monocytes, indicating a possible anti-inflammatory effect [120]. Inhibiting FAAH in monocytes reduces pro-inflammatory cytokines and promotes an anti-inflammatory phenotype [121]. Another study showed that THC and AEA reduced the levels of NO and IL-6 in J774 macrophages treated with LPS. The study also showed that 2-AG slightly increased NO levels and reduced IL-6 like AEA and THC and in a dose dependent manner [122]. Similarly, another group reported that THC and CBD reduce proinflammatory cytokines in alveolar macrophages which was driven by CB2 receptors [123]. Overall, by inhibiting monocyte and macrophage activation, reducing cytokine secretion, and promoting an anti-inflammatory phenotype, these compounds may alleviate the inflammatory processes that contribute to migraine pathophysiology.

## Mast cells and cannabinoids

Mast cells are immune cells that are abundant in connective tissue in the body [124]. Rodent studies have shown that mast cell degranulation is triggered by a tight interaction with dural autonomic and sensory nerves [125]. During a migraine, it is believed that mast cell degranulation increases pro-inflammatory factors, including cytokines, which can activate nociceptors [126]. These mediators are responsible for activating trigeminal afferents, supporting a vicious circle of sensitization proposed to occur during a migraine attack [127]. Surprisingly, unlike rodent cells, human mast cells were reported to not express the full CGRP receptor [128]. However, a later study using a different antibody that is a functional antagonist of the human CGRP receptor, reported antibody binding in primate dural mast cells [17]. The discrepancy is not understood but may reflect differences in epitope availability [11].

Mast cells are also known to produce anti-inflammatory endocannabinoids, including AEA, PEA and 2-AG [129]. In this case, release of a sufficiently high level of endocannabinoids from mast cells could decrease meningeal afferent firing, as shown with exogenous endocannabinoid application [48]. Moreover, mast cells also express both CB1 and CB2 receptors [130]. It has been suggested that the co-expression of both CB receptors may be linked to the complexity of the response in that cell type [130]. The endocannabinoids produced could modulate the activity of the same mast cells or act as immunomodulator in the context of other immune cell types. However, it was shown that most probably only exogenous application of AEA inhibited substance P-induced human mast cell degranulation [131]. Indeed, administration of a pool of different cannabinoids (CP55940, ACEA, WIN552122, and PEA) to mast cell line RBL2H3 activated AKT and ERK phosphorylation via CB2 and suppressed mast cell secretion via CB1 [130]. On the other hand, PEA treatment of mast cell line HMC-1 reduced NGF release not through classical CB receptors but via orphan receptor GPR55 [132]. In contrast to the actions of PEA and the previously mentioned cannabinoids [130], CBD and THC activate mast cell line RBL2H3, raising the question of whether CBD and THC may be acting by different receptors, such as TRPA1 and TRPC1 [133]. In an NTG rat migraine model, methanandamide, a synthetically created stable chiral analog of anandamide, reduced meningeal mast cell degranulation via CB2 receptors [134]. Indeed, neither the CB2 antagonist SR144528 nor the CB1 inverse agonist rimonabant or the TRPV1 agonist capsaicin could reverse this effect [134]. The potential role of mast cells in migraine pathogenesis, combined with the immunomodulatory effects of cannabinoids on their degranulation, warrants further investigation into how these compounds might be used to alleviate migraine symptoms.

## Dendritic cells and cannabinoids

Dendritic cells are another subpopulation of immune cells, residing in the dura mater [135]. These cells are defined as antigen presenting cells because they present the antigens to B and T cells, activating them [136]. Moreover, dendritic cells can facilitate T cell interactions in the central nervous system, contributing to the amplification of local inflammation [137]. Environmental enrichment-mediated changes in dendritic cell exosomes, such as IFNγ-DC-Exos, have shown promise in reducing susceptibility to spreading depression, a potential mechanism underlying migraine, suggesting a therapeutic potential for migraine treatment [138]. These findings highlight the intricate involvement of dendritic cells in the immune responses that may be relevant to migraine.

Dendritic cells express CB1 and CB2 receptors [139], and it has been shown that the activation of CB1 and CB2 receptors on dendritic cells by THC and AEA triggers apoptosis via NF-κB pathway, resulting in immunosuppression [111]. Instead, CBD-treated human dendritic cells increased their performance and release of IL-6, TNF-α, and IL-10 in response to pro-inflammatory LPS [140]. This anti-inflammatory behavior corresponded to less efficient T cells activation [140]. Another study reported that cannabinoid receptor deletion affects dendritic cell development and maturation, leading to altered CD8^+^ T cell responses and influencing immune responses [141]. Synthetic cannabinoids like JWH-015 showed lower HIV replication and an increase in the anti-inflammatory cytokine IL-11 in dendritic cells compared to alcohol and THC, indicating a potential immunomodulatory effect on dendritic cell function [142]. The potential use of cannabinoids for migraine treatment through dendritic cells remains to be investigated.

## B and T cells and cannabinoids

B and T cells are the main cells of the adaptive immune system, driving immune responses against pathogens, maintaining immune homeostasis, and mediating many aspects of autoimmune inflammation [143]. Recent studies have described a copious amount of B cells in rodent meninges [144, 145]. In migraine pathophysiology, circulating lymphocytes, including both B and T cells, have been shown to be decreased in migraine patients compared with controls [19]. The most well-known types of T cells are CD4^+^ and CD8^+^, and within CD4^+^ cells there are regulatory T (Treg) cells and conventional T helper (Th) cells. CD4^+^ Th cells produce IL-10, a primarily anti-inflammatory cytokine which also activates B cells, and plasma levels of IL-10 have been shown to be increased during migraine attacks in patients with migraine without aura, suggesting systematic inflammation in migraine pathogenesis [146]. Like B cells, Treg cells have been shown to be significantly lower in patients with migraine including patients with or without auras and patients with chronic or episodic migraine than healthy controls [147]. A preclinical study demonstrated that repeated NTG treatment lowered the relative levels of Treg cells and that a low-dose of IL-2 was able to expand and activate the Treg cells along with reversing NTG-induced facial hypersensitivity [148]. Of note, the IL-2 effects were also observed in models of post-traumatic headache and medication overuse headache, suggesting that Tregs may be involved in several headache disorders [148].

The CB2 receptor and GPR55, a metabotropic receptor of the ECS, are expressed primarily in cells of the immune system, including B and T cells [149]. Specifically, B cells are the highest CB2-expressing immune cells in humans and express much more CB2 mRNA than CB1 mRNA [150, 151]. Beside expressing cannabinoid receptors, B and T cells also produce both AEA and 2-AG with elevated 2-AG levels in the activated state, leading to a decrease of cytokine release, including IL-6, IL-2, and TNF-α. This effect has been shown to decrease delayed type hypertension via reduction of T-cells proliferation and activation [152]. Moreover, the endocannabinoid 2-AG has been shown to induce chemotaxis of mouse B cells, reversed by CB2-selective antagonist SR144528 [143]. Arvanil, a synthetic capsaicin–anandamide hybrid has been shown to downregulate activation of CD4^+^ T cells [153]. In summary, cannabinoids exert significant immunomodulatory effects on B and T cells by inhibiting cytokine secretion and promoting anti-inflammatory responses, potentially mitigating the neurogenic inflammation that contributes to migraine.

## Glial cells and cannabinoids

Glial cells provide support, protection, and maintenance for neurons, playing essential roles in homeostasis, immune defense, and synaptic regulation [154]. In the CNS, the primary types of glial cells include astrocytes, oligodendrocytes, and microglia [154], while in the PNS, Schwann cells and SGC are the main types [154]. In the TG, small neurons release CGRP, which influences nearby SGC and other neurons [8]. This signaling can stimulate SGC to produce various molecules, including cytokines, which promote inflammation and increase neuronal sensitivity [155]. Specifically, CGRP has been shown to boost the production of IL-1β [156]. Additionally, CGRP release enhances the activity of purinergic receptors on both glial cells and neurons [157, 158], which may further increase CGRP gene transcription. Likewise, ATP released from neurons activates SGC, which then signal back to neurons, amplifying the pro-nociceptive cycle [159].

In the PNS, Christiansen et al. found that CB2 receptors were predominantly expressed in SGC of the murine TG, while CB1 receptors were only on neurons [109] (Fig. 1). Both CB1 and CB2 receptors have been reported on canine Schwann cells [160, 161]. In the CNS, expression of cannabinoid receptors in microglia is closely related to their activation state. Microglial cells in a pro-inflammatory state tend to lower CB1 and CB2 receptor levels, whereas those adopting a repair-promoting phenotype show increased receptor expression [162, 163]. In contrast, 60% of hippocampal astrocyte processes display CB1 receptors on their membranes, with densities comparable to those found in excitatory synapses within the same brain region. On the other hand, there is only limited evidence supporting the presence of CB2 receptors in astrocytes, and it is generally believed that astrocytes in a healthy brain have very low levels of this receptor [164]. Both microglia and astrocytes have the machinery to synthesize and release AEA and 2-AG on demand [165–167].

## Limitations and future perspectives for cannabinoids use in migraine treatment

Overall, the intersection of cannabinoids and immune cells presents a promising but under-investigated strategy for innovative migraine treatments. Indeed, a major limitation in the current landscape of cannabinoid research for migraine is the limited number of published studies and clinical trials. This can partly be attributed to the schedule 1 classification of cannabis products in the US and the illegalization of cannabis world-wide. In the future, this problem should improve as recent legal changes have led to the (re)legalization of cannabis for medical purposes, and the consumption of cannabis products has increased [168].

An area of study with respect to cannabinoids and migraine that needs to be explored is the role of sex. Migraine exhibits a well-known sex disparity in prevalence, with women being 2–3 times more likely to be affected [169]. There are also well documented differences in pain perception and inflammatory response between males and females, which are thought to be influenced by various biological and hormonal factors, including prolactin and sex steroids [170]. For males, elevated testosterone levels have been found to increase pain thresholds, while in females, fluctuations in estrogen levels are associated with heightened pain intensity and perception [171]. Relevant to this review, a study in mice revealed that females are more responsive to macrophage activation, leading to greater pain sensitivity [172]. Similarly, the ECS shows sex-specific differences in receptor expression and endogenous ligand levels. For instance, female rats have higher 2-AG levels in the periaqueductal gray than males [173] and chemotherapy-induced neuropathic pain models reveal distinct ECS enzyme patterns between sexes [174]. Additionally, chronic stress can alter ECS signaling in female rats, potentially worsening pain conditions and reinforcing the need for sex-specific therapeutic strategies [175]. These findings highlight the limitations of a one-size-fits-all approach and the importance of exploring sex-specific mechanisms, particularly in how they may influence the treatment and management of migraine symptoms.

Through targeting various immune cell types such as macrophages, mast cells, dendritic cells, B cells and T cells, cannabinoids can potentially act at multiple receptors to mitigate neurogenic inflammation associated with migraine pathophysiology. However, while using ligands that activate both CB1 and CB2 receptors may enhance therapeutic efficacy, this can also lead to the risk of side effects due to broad distribution of each receptor, especially in the CNS. Furthermore, while cannabinoids may influence the efficacy of CGRP-targeting drugs by modulating the release or action of CGRP in the trigeminovascular system, they can also act at the TRPV1 receptor, which can mask therapeutic effects [107]. Indeed, Christiansen et al. found that the endocannabinoid ACEA acting at CB1 receptors was able to inhibit CGRP release from trigeminal neurons, but only when TRPV1 was inhibited [109]. Consequently, ACEA did not display anti-migraine potential due to its dual agonistic properties at CB1 and TRPV1 receptors [109]. Furthermore, failure of FAAH inhibitor BIA 10-2474 in a phase 1 clinical trial due to serious adverse effects [176], highlights the importance of exercising caution regarding the dosage and selectivity of tested compounds. Large-scale, randomized controlled trials are essential to determine the safety, efficacy, and optimal dosing regimens of cannabinoid-based therapies for migraine patients.

In conclusion, the interactions between cannabinoid receptors and CGRP pathways offers new insights into developing more effective treatments for migraine. In this regard, understanding the intricate interplay between cannabinoids and immune cells in migraine pathology could reveal successful treatments for other disorders characterized by neurogenic inflammation. Hence, novel cannabinoid-based therapies may offer new strategies for effectively managing migraine symptoms and improving the quality of life for individuals affected by this and other debilitating neurological disorders.

## Data Availability

No datasets were generated or analysed during the current study.

## Abbreviations

2-AG: 2-arachidonoylglycerol
ACEA: arachidonyl-2’-chloroethylamide
AEA: anandamide
CBD: cannabidiol
CB1: cannabinoid receptor type 1
CB2: cannabinoid receptor type 2
CFA: complete Freund’s adjuvant
CGRP: calcitonin gene-related peptide
CNS: central nervous system
COX-2: cyclooxygenase-2
ECS: endocannabinoid system
FAAH: fatty acid amide hydrolase
iNOS: inducible nitric oxide synthase
IL: interleukin
LPS: Lipopolysaccharide
MAGL: monoacylglycerol lipase
NO: nitric oxide
NTG: nitroglycerin
PEA: palmitoylethanolamide
PNS: peripheral nervous system
SGC: satellite glial cells
TG: trigeminal ganglia
THC: Δ9-tetrahydrocannabinol
THC-V: tetrahydrocannabivarin
TNF-α: tumor necrosis factor alpha
TRPV1: transient receptor potential vanilloid 1
